# Identification of Diagnostic Markers for Breast Cancer Based on Differential Gene Expression and Pathway Network

**DOI:** 10.3389/fcell.2021.811585

**Published:** 2022-01-12

**Authors:** Shumei Zhang, Haoran Jiang, Bo Gao, Wen Yang, Guohua Wang

**Affiliations:** ^1^ College of Information and Computer Engineering, Northeast Forestry University, Harbin, China; ^2^ Department of Radiology, The Second Affiliated Hospital, Harbin Medical University, Harbin, China; ^3^ International Medical Center, Shenzhen University General Hospital, Shenzhen, China

**Keywords:** breast cancer, KEGG pathway network, SVM, diagnostic markers, gene expression

## Abstract

**Background:** Breast cancer is the second largest cancer in the world, the incidence of breast cancer continues to rise worldwide, and women’s health is seriously threatened. Therefore, it is very important to explore the characteristic changes of breast cancer from the gene level, including the screening of differentially expressed genes and the identification of diagnostic markers.

**Methods:** The gene expression profiles of breast cancer were obtained from the TCGA database. The edgeR R software package was used to screen the differentially expressed genes between breast cancer patients and normal samples. The function and pathway enrichment analysis of these genes revealed significant enrichment of functions and pathways. Next, download these pathways from KEGG website, extract the gene interaction relations, construct the KEGG pathway gene interaction network. The potential diagnostic markers of breast cancer were obtained by combining the differentially expressed genes with the key genes in the network. Finally, these markers were used to construct the diagnostic prediction model of breast cancer, and the predictive ability of the model and the diagnostic ability of the markers were verified by internal and external data.

**Results:** 1060 differentially expressed genes were identified between breast cancer patients and normal controls. Enrichment analysis revealed 28 significantly enriched pathways (*p* < 0.05). They were downloaded from KEGG website, and the gene interaction relations were extracted to construct the gene interaction network of KEGG pathway, which contained 1277 nodes and 7345 edges. The key nodes with a degree greater than 30 were extracted from the network, containing 154 genes. These 154 key genes shared 23 genes with differentially expressed genes, which serve as potential diagnostic markers for breast cancer. The 23 genes were used as features to construct the SVM classification model, and the model had good predictive ability in both the training dataset and the validation dataset (AUC = 0.960 and 0.907, respectively).

**Conclusion:** This study showed that the difference of gene expression level is important for the diagnosis of breast cancer, and identified 23 breast cancer diagnostic markers, which provides valuable information for clinical diagnosis and basic treatment experiments.

## Background

Breast cancer is the phenomenon of runaway proliferation of mammary epithelial cells under the action of various carcinogenic factors. The initial stage of the disease is usually marked by breast mass, nipple discharge, and axillary lymph node enlargement. In advanced stages, cancer cells can spread far away and cause cachexia, which can be manifested by loss of appetite, anorexia, weight loss, fatigue, anemia and fever, and in severe cases can be life-threatening. The incidence of breast cancer ranks first among female malignant tumors. Statistical data show that the incidence of breast cancer in Chinese women is high and tends to be younger (Amer et al., 2020).

Breast gland is the target organ of many endocrine hormones, among which estrone and estradiol are directly related to the occurrence of breast cancer. There are many risk factors for breast cancer, such as early menarche, late menopause age, infertility or late first childbearing age, lack of breast feeding (Breast cancer and breastfeeding, 2002), high estrogen level (Zhou et al., 2014), etc., which are all risk factors for breast cancer. In addition, genetic factors are also high-risk factors for breast cancer. The risk of breast cancer in first-degree relatives is 2–3 times higher than that in the general population (Gage et al., 2012). Some genetic mutations also increase the risk of breast cancer, which has been shown to be associated with estrogen exposure (Cavalieri et al., 2006; Sahu and Pattanayak, 2020). In addition, some physical factors, such as childhood radiation therapy for breast cancer, are also risk factors for breast cancer. Recent studies have found that environmental pollution can also increase the risk of breast cancer (Clapp et al., 2008).

The prognosis of breast cancer is closely related to the stage of disease development, and the earlier the disease is detected, the greater the chance of survival within 5 years. Prognosis is critical for treatment decisions, as minimally invasive treatments such as lumpectomy, radiation or hormone therapy are often offered to patients with a good prognosis. More aggressive treatments, such as more extensive mastectomy and one or more chemotherapy agents, are usually offered to patients with poor prognosis.

Although some progress has been made in the treatment and drug research of breast cancer (Zhuang et al., 2020), studies have shown that due to the lack of understanding of the pathogenesis of this complex disease, there is currently no effective treatment, and the recurrence and death of breast cancer patients are still not effectively controlled (Wapnir et al., 2018). In recent years, with the development of immunology, molecular biology and genomics technology, bioinformatics method has become an important means to study the pathogenesis of diseases, and the identification of valuable biomarkers has become a research focus (Sahu and Pattanayak, 2020; Tanaka et al., 2020; Kanathezath et al., 2021). Therefore, based on bioinformatics analysis methods, pathway and network analysis methods (Yu et al., 2021a) were used to systematically analyze breast cancer related genes, aiming to accurately understand the pathogenesis of breast cancer incidence and development, identify the most important pathogenic genes related to breast cancer, and provide valuable information for clinical diagnosis, treatment and control (Yu et al., 2018; Cao et al., 2021).

Previously, the use of bioinformatics methods to explore differentially expressed genes has made significant progress in pancreatic cancer (Xu et al., 2020), gastric cancer (Cheng et al., 2020; Gu et al., 2021; Yuan et al., 2021), colorectal cancer (Cheng et al., 2021; Zhang and Zhang, 2021), prostate cancer (Rode et al., 2021) and other diseases (Ao et al., 2021; Yu et al., 2021b; Laudisi et al., 2021; Zhang et al., 2021; Zhao et al., 2021). The occurrence and development of breast cancer is a process involving and synergistic action of multiple genes in multiple stages (Kretschmer et al., 2011). Therefore, the differential change of gene expression profile has always been a hot topic in breast cancer research (Manjili et al., 2012). At present, some studies have screened differentially expressed genes in breast cancer patients. Studies have shown that Grb14 is highly expressed in 23.1% of breast cancers, and this high expression is associated with better overall and disease-free survival, and can be used as a better independent prognostic factor (Huang et al., 2013). In adipose tissue of mammary gland, the overexpression of leptin can promote cell proliferation and cancer (Jardé et al., 2011). Abnormal growth factor signaling between stromal cells and epithelial cells can promote malignant cell growth (Wiseman and Werb, 2002; Haslam and Woodward, 2003).

In recent years, many researchers devote themselves to the research of breast cancer and have made great breakthroughs. Staub et al. have shown that patients with a low expression module co-expressed with the WIPF1 gene generally have a good prognosis in three tumor types including colorectal cancer, glioma and breast cancer (Staub et al., 2009). Protease has also been studied as a biomarker for prognosis and diagnosis of breast cancer (Jardé et al., 2011). Song et al. also demonstrated that the expression of transforming acid curly spiral egg (TACC3) gene is an independent prognostic factor in breast cancer patients (Song et al., 2018).

This study aims to identify diagnostic markers of breast cancer by differential expression genes screening and pathway networks constructing. The gene expression profile was used to screen the genes related to breast cancer, pre-screen the diagnostic markers of breast cancer, and analyze the function of these genes. The potential breast cancer markers were further explored through the construction of KEGG pathway network, and reliable breast cancer diagnostic markers were screened by combining with differentially expressed genes. The diagnostic prediction model was constructed using these markers, and the predictive power of the model was verified. In addition, these markers are documented to provide targets and references for clinicians as well as biological experimentalists.

## Methods

### Data Acquisition and Processing

In this study, expression profile data of breast cancer were obtained from The Cancer Genome Atlas (TCGA) database (Cancer Genome Atlas Research et al., 2013; Li et al., 2020). The TCGA database is one of the most ambitious and valuable cancer genomics projects currently under way. It is a joint initiative of the National Institute of Cancer Research and the National Human Genome Research Institute. It had the molecular signatures of more than 20,000 primary tumors and matched normal samples covering 33 cancer types. It contains clinical data on a variety of human cancers, genomic mutations, mRNA expression, miRNA expression, methylation, and other data, which is a very important source of information for cancer researchers (Tang et al., 2018; Tian and Wang, 2021; Zhu et al., 2021). These data have improved our ability to diagnose, treat and prevent cancer and will continue to be publicly available to anyone in the research community.

The level 3 gene expression data of RNA-SeqV2 in breast cancer were downloaded from the TCGA database and included 1218 samples, including 1104 breast cancer samples and 114 normal control samples, containing 20,530 genes. The gene expression profile was measured experimentally using the Illumina HiSeq 2000 RNA Sequencing platform by the University of North Carolina TCGA genome characterization center. This dataset shows the gene-level transcription estimates, as in log2(*x*+1) transformed RSEM normalized count. Genes are mapped onto the human genome coordinates using UCSC Xena HUGO probeMap.

### Screening of Differentially Expressed Genes

In our study, the potential related genes of breast cancer were obtained by screening the differentially expressed genes. Differentially expressed genes were screened using edgeR software package. EdgeR software package is a Bioconductor software package for differential expression analysis of RNA-seq expression profiles with biological replication, which is used to identify differential expressions or differential markers using read counts from different technology platforms (including RNA-Seq, ChIP-seq, ATAC-seq, Bisulfite-seq, SAGE, etc.). It implements a range of statistical methodology based on the negative binomial distributions, including empirical Bayes estimation, exact tests, generalized linear models and quasi-likelihood tests (Robinson et al., 2010).

The screening principle of differentially expressed genes was based on the Fold Change (FC) and Pvalue of breast cancer genes between breast cancer patients and the control group. Set filter conditions when Pvalue <0.05, and the Fold Change of gene expression value was satisfied with FC > 2, or FC < 0.5, the genes that satisfy both conditions were identified as differentially expressed gene.

These differentially expressed genes were displayed using heat map and volcano plot. The heat map was plotted using gplots R package and the volcano plot was plotted using ggplot2 R package.

### Biological Functions and Pathways Enrichment Analysis of Differentially Expressed Genes

In this study, using DAVID (database for Annotation, Visualization and Integrated Discovery) (Huang et al., 2009a; Huang et al., 2009b), a database used for annotation, visualization and integration of discoveries, we conduct a GO (Gene Ontology) biological functions enrichment analysis and a KEGG (Kyoto Encyclopedia of Genes and Genomes) pathways enrichment analysis towards the list of differentially expressed genes, with *p* controlled within 0.05, which could find out the biological characteristics and pathways related.

DAVID is a bioinformatic database that combines information tools to provide a structured and complete description of biological functions for a large number of genes or proteins and help users obtain useful biological information.

### Construction of KEGG Pathway Gene Interaction Network and Extraction of Key Genes

KEGG (Kyoto Encyclopedia of Genes and Genomes) is a database tool for understanding the advanced functions and availability of biological systems (such as cells, organisms, and ecosystems) based on information at the genome and molecular levels (Kanehisa and Goto, 2000). It is a computer representation of a biological system formed by molecular blocks of genes, proteins and chemicals that are integrated into molecular wiring diagrams of an information system of interactions, reactions and relationships. It also contains disease and drug information as disturbances to biological systems.

In this study, the KGML which were organized as XML files of significant pathways obtained from the previous enrichment analysis was downloaded from the KEGG website. Extract relation, entry and group in these XML files using “XML” R package. The entry element contains information about a node of the pathway. Relationship between two proteins (gene products) or two KOs (ortholog groups) or protein and compound, which is indicated by an arrow or a line connecting two nodes in the KEGG pathways. Group stands for complex of KOs group. The types associated between nodes include ECrel, PPrel, GErel, PCrel and maplink. Respectively represent enzyme-enzyme relation, protein-protein interaction, gene expression interaction, protein-compound interaction and link to another map. We integrate the real protein-protein interaction by connecting these three types of files and construct the KEGG path gene interaction network, and analyze the topology properties of the network. The network was analyzed and visualized using Cytoscape software (Shannon et al., 2003). Cytoscape is an open-source software platform that allows users to visualize molecular interaction networks and biological pathways and integrate these networks with annotated information, gene expression profiles and other data.

The size of a node in the network is expressed by the degree of the node. The gene with a large degree of node is called Hub gene in the network. The larger its value is, the more edges are connected to the node, and it may play a more important role in maintaining the overall structure of the network. Its change may affect more genes that interact with it. Therefore, in this study, genes with a degree greater than 30 were extracted from the network as key genes affecting breast cancer.

### Literature Mining Confirms the Genes Screened

Next, we take the intersection of the obtained differentially expressed genes and the Hub genes obtained in the previous step to obtain a narrow and reliable biomarker list for breast cancer diagnosis.

To test whether the biomarkers screened in our study are indeed associated with breast cancer, we use PubMed (www.pubmed.gov) to conduct a literature review, and analyze whether the genes are indeed related to breast cancer in previous reports, so as to prove that the screening of tumor-related genes using our methods is effective. PubMed (White, 2020) is a widely used search engine, built and maintained by the National Biotechnology Information Center (NCBI) of the National Library of Medicine (NLM), which can provide more than 28 million academic biomedical publications.

This method is simple and feasible. The selected genes and breast cancer will be searched as the keywords in the literature database, and then consult the literature to see if there is a strong or weak relationship between the screened genes and the occurrence and development of breast cancer.

### The Construction of Diagnosis and Prediction Model of Breast Cancer

According to the expression profile data of breast cancer, the corresponding expression values of 23 breast cancer diagnostic markers obtained in the previous step were found, and the expression profile data of these 23 genes were obtained. To verify the accuracy of the model, cancer patients and normal control samples in this dataset were randomly divided into two sets, one for training set and another for test set. The principle of division was to ensure that the proportion of cancer patients and normal control samples in each set was the same. The training set included 609 samples, including 552 samples from breast cancer patients and 57 normal control samples. The validation dataset consisted of 609 samples, including 552 samples from breast cancer patients and 57 normal controls.

Next, we use the training set samples to build the support vector machine model. Support Vector machines (SVM) is a kind of dichotomous classification model. Its basic model is a linear classifier, which defines the maximum interval in the feature space, which is the biggest difference between it and perceptron. The SVM learning strategy is to maximize the interval, which can be formalized as a problem for solving convex quadratic programming.

The purpose of the SVM model is to find the maximum distance between each sample point and the hyperplane, that is, to find the hyperplane with the largest interval. Any hyperplane can be described by the following linear equation:

$$w^{T}x+b=0$$ (1)

Now let’s start to calculate the interval. In fact, the interval is equal to the projection of the difference of two heterogeneous support vectors on *w*:

$$\gamma =\frac{\left({\vec{x}}_{+}-{\vec{x}}_{-}\right)\cdot {\vec{w}}^{T}}{\mathrm{||w||}}=\frac{{\vec{x}}_{+}\cdot {\vec{w}}^{T}-{\vec{x}}_{-}\cdot {\vec{w}}^{T}}{\mathrm{||w||}}$$ (2)

*x* satisfy:

$$\{\begin{array}{c} 1^{*}\left(w^{T}x_{+}+b\right)=1,y_{i}=+1\\ -1^{*}\left(w^{T}x_{-}+b\right)=1,y_{t}=-1 \end{array}$$ (3)

Get:

$$\gamma =\frac{1-b+\left(1+b\right)}{\left\|w\right\|}=\frac{2}{\left\|w\right\|}$$ (4)

After the interval is solved, the idea of SVM is to maximize the interval, so:

$$\max_{v,b}\frac{2}{\left\|w\right\|},s\mathrm{.t}.\;y_{i}\left(w^{T}x_{i}+b\right)\geq 1\left(i=1,2,\ldots ,m\right)$$ (5)

max and min to obtain the optimization problem:

$$\min_{w,b}\frac{1}{2}{\left\|w\right\|}^{2}\text{,}\;s\mathrm{.t}.\;y_{i}\left(w^{T}x_{i}+b\right)\geq 1\left(i=1,2,\ldots ,m\right)$$ (6)

The classification model was tested internally using a tenfold cross validation method, and the model was tested externally using test sets to verify the accuracy of the model in classifying new patients. The performance of the model was also measured using sensitivity and specificity (Cheng et al., 2018; Tao et al., 2020; Zhao et al., 2020).

## Results

### Acquisition of Differentially Expressed Genes in Breast Cancer

**FIGURE 1:**
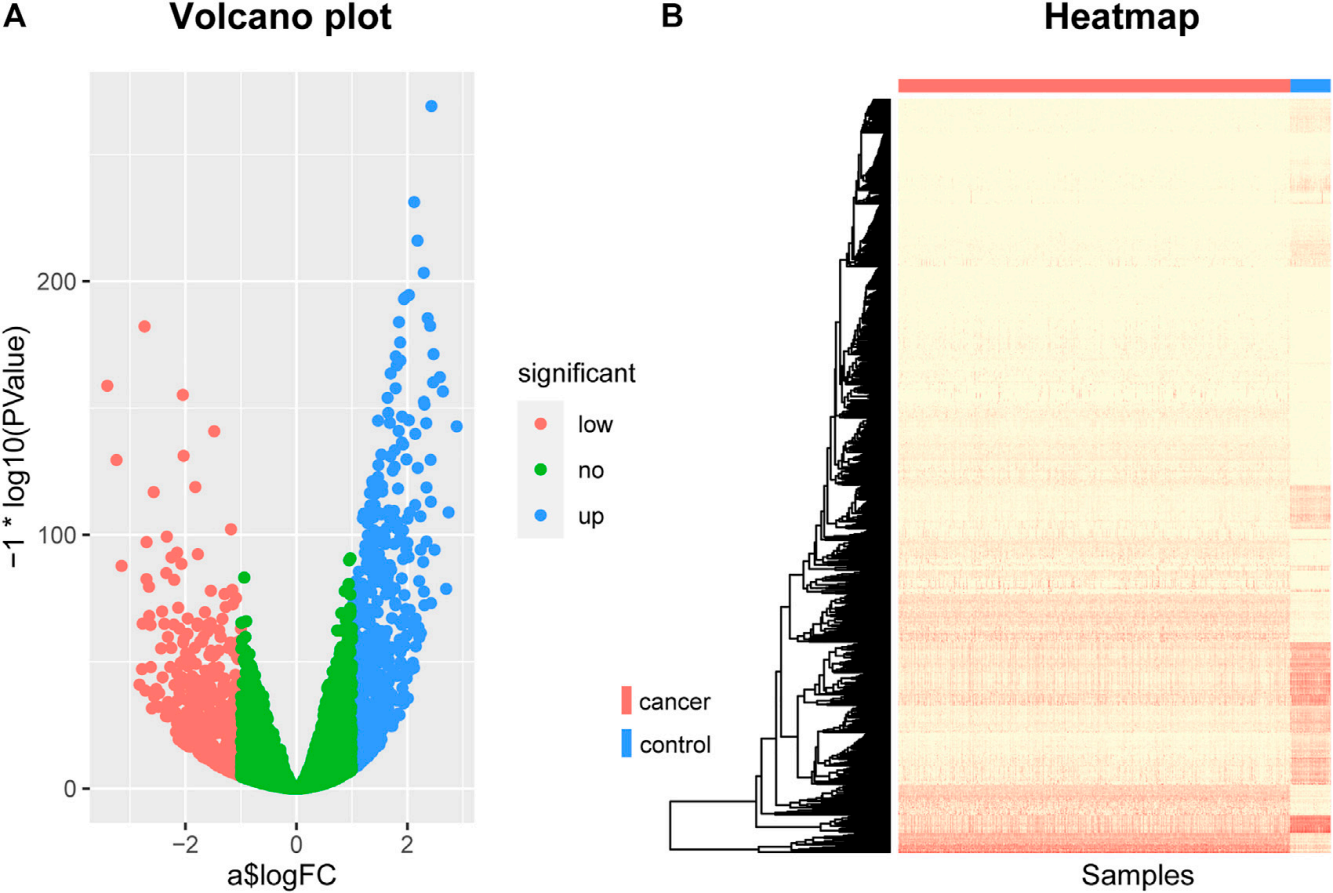
The Volcano plot and Heat map of differentially expressed genes. **(A)** The Volcano plot of differentially expressed genes, where the red dots represent the genes that are down-regulated in breast cancer, the blue dots represent the genes that are up-regulated in breast cancer, and the green dots represent the genes that are not significantly different. **(B)** The Heat map of differentially expressed genes. The rows represent differentially expressed genes and the columns represent patients, where the red bar represents breast cancer patients and the blue bar represents normal control samples.

By screening for differentially expressed genes, we obtained 1060 genes that were differentially expressed between breast cancer patients and normal control samples. These genes can be seen as potentially related to breast cancer. Among them, 516 were down-regulated genes and 544 were up-regulated genes. They were displayed using a volcano plot (Figure 1A), where the red dots represent the genes that are down-regulated in breast cancer, the blue dots represent the genes that are up-regulated in breast cancer, and the green dots represent the genes that are not significantly different.

In addition, we also used a heat map to show the expression levels of these differentially expressed genes in cancer and normal samples (Figure 1B). The rows in the figure represent differentially expressed genes and the columns represent patients, where the red bar represents breast cancer patients and the blue bar represents normal control samples. As can be seen from this figure, there are indeed significant differences in these genes between breast cancer patients and normal control samples, which can clearly distinguish cancer patients from normal control samples. This suggests that the genes we screened are indeed associated with breast cancer.

### Pathways and Biological Functions Differentially Expressed Genes Involved

**FIGURE 2:**
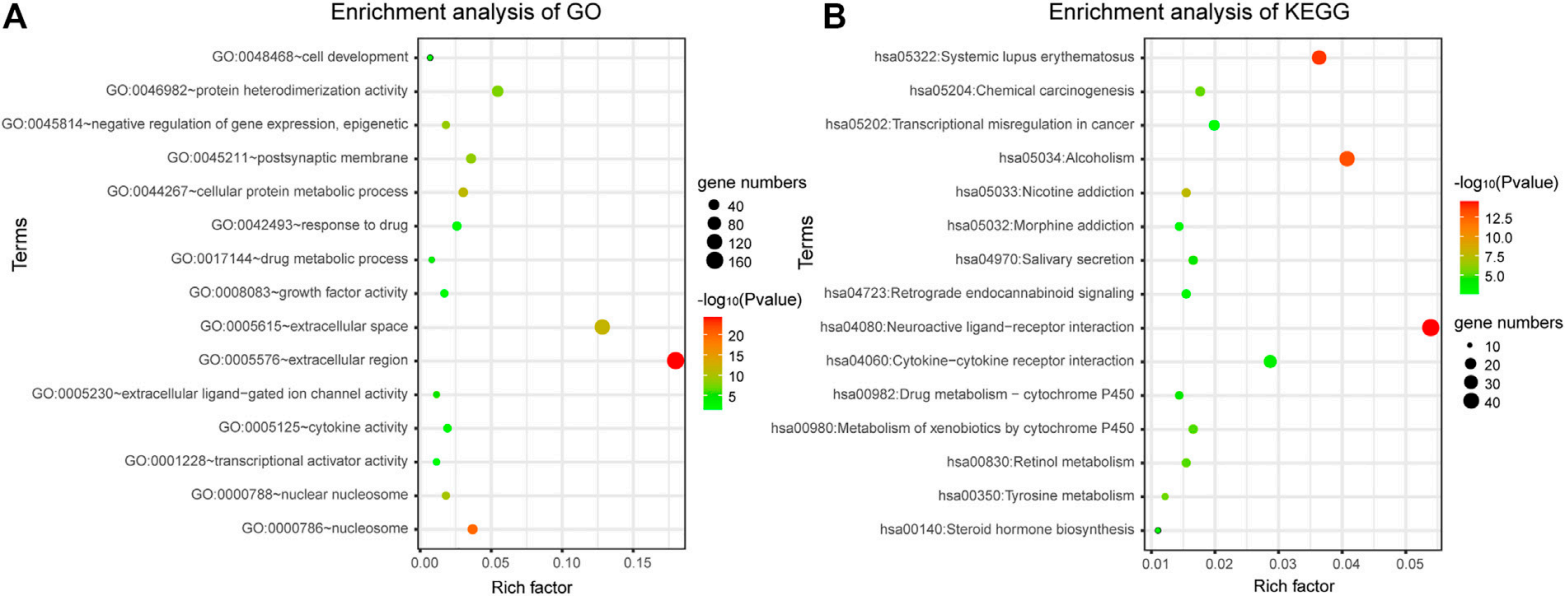
Gene enrichment analysis. **(A)** Gene enrichment analysis of Gene Ontology. **(B)** Gene enrichment analysis of KEGG.

DAVID bioinformatics tool was employed to complete the Gene Ontology and KEGG pathway enrichment analysis. From the result of enrichment, we can see that the differentially expressed genes are involved in a number of cancer-related biological functions, such as cellular protein metabolic process, negative regulation of gene expression, epigenetic, response to drug, cell development, transcriptional activator activity, RNA polymerase II transcription regulatory region sequence-specific binding et al. (Figure 2A). Through the bubble diagram, it can be seen intuitively that the GO function and KEGG pathway enriched by the 1060 differentially expressed genes. It is significant that these genes annotate the transcriptional misregulation in cancer pathway, suggesting that these genes may be closely related to cancer development and may serve as potential biomarkers for breast cancer.

In addition, a total of 28 significantly enriched pathways were identified (Table 1; Figure 2B), including Neuroactive ligand-receptor interaction, Systemic lupus erythematosus, Chemical carcinogenesis, Metabolism of xenobiotics by cytochrome, Drug metabolism—cytochrome P450, Transcriptional misregulation in cancer, cAMP signaling pathway et al. This suggests that the occurrence and development of breast cancer is a complex physiological process, with abnormal functions in a variety of pathways.

**TABLE 1:** KEGG pathways enriched by differentially expressed genes in breast cancer.

Term	Count	*p v*alue
hsa04080: Neuroactive ligand-receptor interaction	49	2.82E-15
hsa05322: Systemic lupus erythematosus	33	1.72E-14
hsa05034: Alcoholism	37	7.54E-14
hsa05033: Nicotine addiction	14	2.33E-08
hsa00350: Tyrosine metabolism	11	3.84E-06
hsa05204: Chemical carcinogenesis	16	5.16E-06
hsa00830: Retinol metabolism	14	8.86E-06
hsa00980: Metabolism of xenobiotics by cytochrome P450	15	9.57E-06
hsa04970: Salivary secretion	15	5.62E-05
hsa00982: Drug metabolism - cytochrome P450	13	8.52E-05
hsa04060: Cytokine-cytokine receptor interaction	26	2.69E-04
hsa04723: Retrograde endocannabinoid signaling	14	0.0011
hsa05032: Morphine addiction	13	0.0014
hsa00140: Steroid hormone biosynthesis	10	0.0017
hsa04727: GABAergic synapse	12	0.0025
hsa05202: Transcriptional misregulation in cancer	18	0.0028
hsa04972: Pancreatic secretion	12	0.0050
hsa04974: Protein digestion and absorption	11	0.0097
hsa04975: Fat digestion and absorption	7	0.0104
hsa00591: Linoleic acid metabolism	6	0.0117
hsa03320: PPAR signaling pathway	9	0.0150
hsa04024: cAMP signaling pathway	18	0.0152
hsa04976: Bile secretion	9	0.0176
hsa04724: Glutamatergic synapse	12	0.0217
hsa04913: Ovarian steroidogenesis	7	0.0297
hsa04950: Maturity onset diabetes of the young	5	0.0347
hsa00010: Glycolysis/Gluconeogenesis	8	0.0422
hsa00910: Nitrogen metabolism	4	0.0462

### Acquisition of Potential Diagnostic Markers for Breast Cancer

**FIGURE 3:**
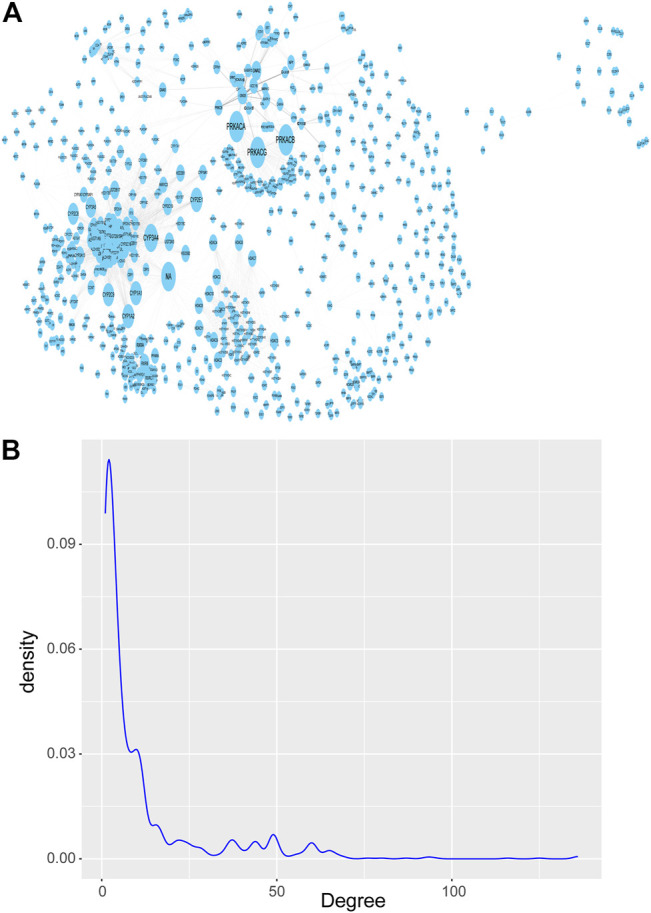
KEGG pathways network of gene-gene interaction. **(A)** KEGG pathways network, which contains 1277 nodes and 7345 edges, and the size of the nodes is represented by the degree. **(B)** Probability density distribution of nodes degree in the network, which conforms to the power law distribution.

Next, we downloaded the pathways with significant enrichment of differentially expressed genes obtained above from the KEGG website. Then a network is constructed by integrating the real gene interaction information from the KEGG pathways. The network contains 1277 nodes and 7345 edges, and the size of the nodes is represented by the degree (Figure 3A). Of the 1277 nodes (genes), 175 were differentially expressed genes (a total of 1060) previously screened between breast cancer and normal control samples, and the remaining 1102 genes were obtained by pathway enrichment analysis. If the key genes (nodes with large degree) are removed, the stability of the KEGG pathway gene interaction network will be seriously threatened, and the network topology will be lax. Therefore, although the nodes with large degree only account for a small part of the nodes in the network, which conforms to the power law distribution of the degree distribution of the biomolecular network (Figure 3B), they are essential key nodes.

The nodes with large degree in the network are called Hub nodes. These nodes are generally the key nodes in the network, because their changes may affect more genes that interact with them. We tend to select the top 10% nodes in the network as Hub nodes. However, under this threshold, there are many nodes with the same degree and their degree is 38. Therefore, in order to ensure less omission of breast cancer diagnostic markers, we select nodes with a degree greater than 30 on the basis of this threshold. So, we filtered the degree attribute according to the attribute table exported after topology analysis and set the filter condition as degree >30, 154 genes, known as Hub genes, were identified as candidate genes potentially associated with breast cancer.

In this study, the common gene set of the previously obtained differentially expressed genes in breast cancer and key genes in the pathway network were selected, including 23 genes (Table 2). As candidate markers for the diagnosis of breast cancer, to ensure the accuracy of these markers.

**TABLE 2:** The candidate diagnostic markers for breast cancer.

Gene	Degree	Pvalue	Gene	Degree	*p v*alue
ADCY8	37	1.41E-32	CYP2C19^a^	56	1.74E-10
ADH1A^a^	65	1.50E-167	CYP3A4^a^	116	1.66E-66
ADH1C^a^	65	3.22E-87	CYP3A7^a^	43	1.59E-21
ADH4	65	4.01E-152	GNG13	44	1.61E-88
ADH6	65	1.43E-19	GNGT1	44	3.49E-22
ADH7	65	1.45E-28	GSTA1^a^	49	2.20E-66
AKR1C4^a^	25	4.83E-10	HSD3B1	52	3.17E-17
ALDH3A1^a^	38	2.24E-70	HSD3B2	52	4.44E-53
CYP1A2^a^	93	3.34E-23	RXRG^a^	64	1.15E-66
CYP2A13	58	1.23E-09	UGT1A7	60	9.17E-43
CYP2B6^a^	62	3.57E-25	UGT2B28	60	2.46E-44
CYP2C18^a^	55	6.14E-17	—	—	—

a These genes have been documented to be associated with breast cancer.

### Breast Cancer Related Genes Were Confirmed by Literature Review

Next, to verify that our method of screening for biomarkers for breast cancer is reliable. We selected the genes for literature mining and verification. To see if there is any evidence in the literature that these genes are indeed involved in the development and progression of breast cancer.

After the intersection of the two sets of genetic data sets, 23 diagnostic markers of breast cancer were obtained. After literature mining in PubMed literature retrieval system, we found that some of these 23 genes have been confirmed to be related to breast cancer, including 12 genes, ADH1A, ADH1C, AKR1C4, ALDH3A1, CYP1A2, CYP2B6, CYP2C18, CYP2C19, CYP3A4, CYP3A7, GSTA1, RXRG. For example, THE TT genotype of CYP3A4 polymorphism is associated with increased risk of breast cancer (Liu et al., 2019). RXRG protein is an independent predictor of breast cancer specific survival and distant metastasis-free survival (Joseph et al., 2019). ADH1A has been found to be a potential biomarker for diagnosis, treatment and prognosis of breast cancer (Wu and Yu, 2021). It is worth noting that the remaining genes, such as GNGT1 and UGT1A7, have not been documented to be associated with breast cancer. However, the literature has linked these genes to other cancers. Therefore, the prediction of the correlation between these genes and breast cancer will provide clinicians and biological experimentalists with targets and references for future research directions.

### Construction and Prediction of Breast Cancer Diagnostic Model

According to the expression profile data of breast cancer, the corresponding expression profile data of 23 diagnostic markers of breast cancer obtained in the previous step were obtained. Cancer patients and normal control samples were divided into training sets and test sets. The proportion of breast cancer patients and normal control samples in both datasets was also ensured to be the same. The training set included 609 samples, including 552 samples from breast cancer patients and 57 from normal control samples; the validation dataset also consisted of 609 samples, including 552 samples from breast cancer patients and 57 from normal controls.

**FIGURE 4:**
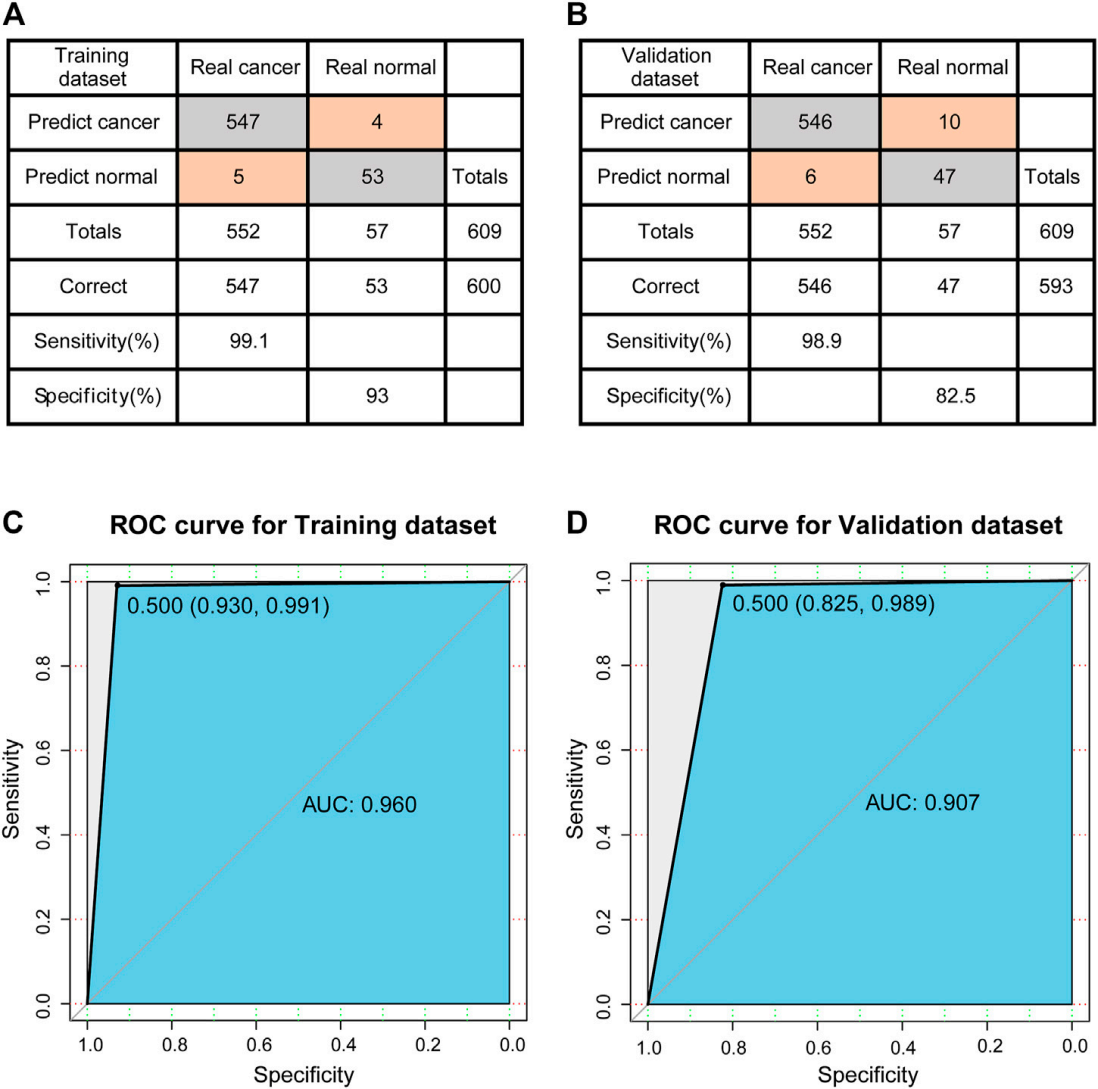
Evaluation of classification models. **(A)** The confusion matrix of Training dataset. **(B)** The confusion matrix of Validation dataset. **(C)** ROC curve of Training dataset. **(D)** ROC curve of Validation dataset.

The training set was used to construct the classification model of support vector machine. The accuracy of the model was evaluated using a tenfold cross validation method. The confusion matrix was shown in Figure 4A. According to the matrix, 600 out of 609 samples were classified correctly, and the classification accuracy was 98.5%. The sensitivity and specificity of the model were 99.1 and 93%, respectively. The ROC curve (receiver operating characteristic curve) of the model is shown in Figure 4C, and the AUC reaches 0.960.

Next, the established model is used to predict the test set to test the prediction ability of the model. The confusion matrix is shown in Figure 4B, 593 out of 609 samples in test set are correctly classified, and the classification accuracy is 97.4%. The sensitivity and specificity of the model were 98.9 and 82.5%, respectively. Compared to the training set, there was a decrease in specificity and a certain increase in the misclassified normal control samples. The ROC curve of the model is shown in Figure 4D, and the AUC reaches 0.907. In other words, for new patients, once we have data on the expression levels of these 23 genes, we can use the classification model constructed in this study to predict whether they are likely to develop breast cancer.

These results indicate that the diagnostic prediction model constructed in this study can effectively distinguish between breast cancer patients and normal control population, and these 23 genes can be used as reliable biomarkers for breast cancer diagnosis, but further experiments are still necessary. Especially for genes that have not been reported in the literature. In addition, we analyzed the biological functions of these 23 diagnostic markers and found that they were involved in many cancer-related biological processes and pathways, such as chemical carcinogenesis, drug metabolism, xenobiotic metabolic process, metabolic pathways, oxygen binding etc.

## Discussion

Breast cancer is a heterogeneous cancer with the highest incidence among women in the world, which is a serious threat to women’s health. The occurrence of breast cancer is a complex biological process involved and regulated by multiple genes, and the difference of gene expression levels in tumor cells of different patients determines the different treatment and prognosis of patients (Saad et al., 2010). Therefore, to explore the characteristic changes of breast cancer from the level of genes and the discovery of biomarkers of breast cancer diagnose will play an important role in guiding the treatment of breast cancer.

Advances in high-throughput sequencing technology have made it easier for researchers to understand how diseases occur and develop at the genome-wide level. RNA-Seq refers to transcriptome sequencing technology, which is a high-throughput sequencing technology to reflect the expression level of mRNA, small RNA and noncoding RNA or some of them by determining their sequences with high-throughput sequencing technology. TCGA is an open and free database resource that integrates multiple cancer data types. A large sample size of RNA-Seq data for breast cancer can be obtained. In this study, the Level 3 gene expression data of RNA-SeqV2 in 1104 breast cancer tumor samples and 110 normal control samples adjacent to cancer were downloaded from the TCGA database, and the genetic characteristics of breast cancer were analyzed at the whole genome level using these data. Identify genes that are differentially expressed in breast cancer and molecular markers for cancer diagnosis.

In this study, a total of 1060 differentially expressed genes were screened from the tumor and normal samples of breast cancer by using edgeR R package, of which 544 genes were up-regulated and 516 were down-regulated in the cancer samples. Through the enrichment analysis of GO function, it was found that these genes were mainly enriched in biological processes such as cellular protein metabolic process, negative regulation of gene expression, epigenetic, response to drug, cell development, transcriptional activator activity, RNA polymerase II transcription regulatory region sequence-specific binding et al. In addition, KEGG pathway enrichment analysis of these genes showed that they were significantly enriched in 28 pathways such as Neuroactive ligand-receptor interaction, Systemic lupus erythematosus, Chemical carcinogenesis, Metabolism of xenobiotics by cytochrome, Drug metabolism - cytochrome P450, Transcriptional misregulation in cancer, cAMP signaling pathway et al. By integrating the interaction information of genes in these pathways, a KEGG pathway network was constructed, and the hub nodes in the network were extracted, and 154 candidate genes potentially related to breast cancer were obtained. By integrating with the list of differentially expressed genes, 23 potential diagnostic markers of breast cancer were finally obtained. Some of these genes have been shown to play important roles in the development of breast cancer, which confirms the reliability of the results of this study. However, the remaining genes have not been studied in relation to the risk of breast cancer. They may be new diagnostic factors or risk genes for breast cancer, so further analysis and experimental confirmation of these genes are necessary.

Using gene expression profile of the 23 diagnostic marker genes, we constructed the breast cancer diagnosis prediction model based on SVM classifier, and analyzed the model prediction ability. The results proved that the model can effectively distinguish breast cancer patients and normal people, and further identifying these 23 genes can be used as diagnostic markers of breast cancer. It provides targets and reference for clinical doctors and biological experimentalists to treat breast cancer. It is worth noting that many of the 23 genes come from the same gene family, and further research is needed to determine whether there is redundant information between them. In addition, the methods in this paper can also be applied to other patient data to guide the diagnosis and treatment of cancer patients.

In this study, bioinformatics research methods were used to systematically and comprehensively analyze the differentially expressed genes related to breast cancer, and explore the pathogenesis, therapeutic targets and target prediction of breast cancer.

In conclusion, the use of computer technology and mathematical modeling and other methods can effectively analyze the differential expression genes related to breast cancer, so as to understand the pathogenesis of breast cancer and predict the prevention and treatment targets. It can also effectively solve the problems in biomedical science, and further provide meaningful information for future experimental research.

## Data Availability

The datasets presented in this study can be found in online repositories. The names of the repository/repositories and accession number(s) can be found in the article/Supplementary material.
